# Host Cell Transcriptome Profile during Wild-Type and Attenuated Dengue Virus Infection

**DOI:** 10.1371/journal.pntd.0002107

**Published:** 2013-03-14

**Authors:** October M. Sessions, Ying Tan, Kenneth C. Goh, Yujing Liu, Patrick Tan, Steve Rozen, Eng Eong Ooi

**Affiliations:** 1 Program in Emerging Infectious Disease, Duke-NUS Graduate Medical School, Singapore; 2 Centre for Computational Biology, Duke-NUS Graduate Medical School, Singapore; 3 Computational Systems Biology, Singapore-MIT Alliance, National University of Singapore, Singapore; 4 Program in Cancer and Stem Cell Biology, Duke-NUS Graduate Medical School, Singapore; University of California, Berkeley, United States of America

## Abstract

Dengue viruses 1–4 (DENV1-4) rely heavily on the host cell machinery to complete their life cycle, while at the same time evade the host response that could restrict their replication efficiency. These requirements may account for much of the broad gene-level changes to the host transcriptome upon DENV infection. However, host gene function is also regulated through transcriptional start site (TSS) selection and post-transcriptional modification to the RNA that give rise to multiple gene isoforms. The roles these processes play in the host response to dengue infection have not been explored. In the present study, we utilized RNA sequencing (RNAseq) to identify novel transcript variations in response to infection with both a pathogenic strain of DENV1 and its attenuated derivative. RNAseq provides the information necessary to distinguish the various isoforms produced from a single gene and their splice variants. Our data indicate that there is an extensive amount of previously uncharacterized TSS and post-transcriptional modifications to host RNA over a wide range of pathways and host functions in response to DENV infection. Many of the differentially expressed genes identified in this study have previously been shown to be required for flavivirus propagation and/or interact with DENV gene products. We also show here that the human transcriptome response to an infection by wild-type DENV or its attenuated derivative differs significantly. This differential response to wild-type and attenuated DENV infection suggests that alternative processing events may be part of a previously uncharacterized innate immune response to viral infection that is in large part evaded by wild-type DENV.

## Author Summary

Dengue is the most common insect-borne viral disease globally. The continued absence of an effective therapy stems from an incomplete understanding of disease pathogenesis, of which the host response to infection is thought to play a central role. While previous studies have described the changes in total gene expression with dengue virus infection, they have not been able to provide any information on the subtle variations of the host RNA. These variations lead to the production of gene isoforms that can have a profound effect on gene function. In the current study, we have used the newly developed technique of RNA sequencing to more accurately interrogate the variations in the host RNA after infection with a wild-type dengue virus or its attenuated derivative. Findings from this study show that there is an extensive amount of previously uncharacterized variation in host RNA response to dengue infection. The response to infection with the wild-type dengue also differs significantly from infection with the vaccine strain. This suggests that variations in the host RNA comprise a part of the host response to viral infection that is in large part evaded by wild-type dengue viruses.

## Introduction

Dengue viruses 1–4 (DENV1-4) are the world's most prevalent arthropod-borne viruses [1]. DENVs are responsible for an estimated 50–100 million cases of debilitating or life-threatening infection every year and an estimated 2.5 billion people in over 100 endemic countries are at risk of infection [1], [2]. The economic impact of DENVs has been estimated to be as high, if not higher than other major global health menaces such as malaria, tuberculosis, hepatitis, bacterial meningitis and others [3]–[8]. Despite the considerable health and economic impact, there are as yet no licensed vaccines or antiviral drugs to combat DENVs and an incomplete understanding of the biology of DENV infection has hampered progress on both of these fronts.

Given the limited coding capacity of their ∼11 kb RNA genome, DENVs must parasitize the host cell machinery to complete their life cycle. At the same time, these viruses must effectively evade or suppress the host responses that act to restrict their replication [9]–[11]. This interplay between host and virus and the effect it has on host gene expression has been described previously [12]–[29]. Largely uncharacterized, however, is whether the transcriptional start site (TSS) and post-transcriptional variations of host RNA, leading to the production of different gene isoforms, may play a role in DENV infection. Differential RNA processing is known to be a major factor underlying cellular and functional complexity [30], [31]. In order to interrogate TSS and post-transcriptional RNA variations across the entire genome in response to DENV infection, we harnessed the power of RNA sequencing (RNAseq). RNAseq is a recently developed approach to transcriptome profiling that permits a precise quantification of RNA levels and their alternatively processed variants by means of high throughput, massively parallel sequencing and subsequent mapping of the resultant short sequence fragments onto a reference genome [32], [33].

We utilized two strains of DENV1 in our RNAseq study to identify strain-specific TSS and post-transcriptional variations in response to infection. The first strain, DENV1-16007, was isolated from the serum of a patient in Thailand in 1964. The second strain of DENV1 used in this study is an attenuated derivative of DENV1-16007. This attenuated virus, DENV1-PDK13 was passaged 13 times in primary dog kidney cells and was shown to be immunogenic but minimally reactogenic in human volunteers [34], [35]. Our RNAseq data indicate that significant differences exist between these two strains of DENV1, not only at the transcript level but also at the level of alternative splicing. Similar trends were observed in RNAseq of an additional two low-passaged DENV1 clinical isolates. These findings suggest that subversion of the host response includes TSS and post-transcriptional modification and is part of the mechanism of virulence. These findings also suggest that variations in the viral genome can have a profound effect in modifying host response to infection.

## Materials and Methods

### Cells and virus stock

HuH7, C6/36 and BHK-21 cells were purchased from the American Type Culture Collection (ATCC) and cultured according to ATCC recommendation. DENV1 strains 16007 and PDK13 were obtained from the Division of Vector-borne Diseases, Centers for Disease Control and Prevention. Sequence analysis in our laboratory indicates that these strains match the published sequences for these viruses (GenBank accession numbers AF180817.1 and AF180818.1, respectively). These viruses were amplified three times in C6/36 cells prior to use in the current study. Additionally, the DENV1 clinical isolates EDEN3300 and SL107, which were obtained from previously reported studies [36], [37] and passaged in C6/36 cells for <5 times were also included. The supernatant of all virus cultures were harvested six days post infection, clarified by centrifugation at 450× g for 10 min at 4°C, filtered and concentrated by centrifugation at 30,000× g for 3 hrs at 4°C. Virus pellets were re-suspended in DMEM medium with 2% FBS (Invitrogen) and stored at −80°C until use. Infectious titer was determined by plaque assay as described previously [38].

### Virus infection in HuH7 cells

HuH7 cells were seeded at 3×10^6^ per flask in 25 mm flasks and incubated at 37°C for 24 hrs before infecting at a MOI of 20 with each of the DENV strains for 1 hr at 37°C/5% CO_2_, with gentle rocking every 15 min. The cells were then washed thoroughly and replaced with DMEM medium supplemented with 2% FBS and incubated for 20 hrs.

### Immunofluorescence assay

Immunofluorescence assay was conducted according to a previously described method [39]. Briefly, the cells from the virus culture were washed once and re-suspended with PBS, and spotted onto a Teflon coated glass slide, air dried and then immersed in 80% acetone for 10 min. The slide was rinsed with PBS and air-dried. 2 µl antibody against prM protein (2H2 monoclonal antibody) was added onto each well, incubated at 37°C for 45 min in a humidified chamber, and washed twice with PBS before drying. FITC-conjugated goat anti-mouse IgG were diluted 1∶30 with 0.1% Evan's Blue and 2 µl was added onto each well. Slides were then incubated at 37°C for 45 min in the humidified chamber and then washed twice with PBS. Slides were dried and mounted with buffered glycerol before imaging under a fluorescent microscope.

### Generation of whole-transcriptome cDNA library

Polyadenylated mRNA was isolated from HuH7 cells by three rounds of selection with the Dynabeads mRNA Direct Kit (Invitrogen) and assessed by electrophoresis on the Bioanalyzer 2100 (Agilent) for quality evaluation. For the RNAseq sample preparation, the NEBNext mRNA Sample Prep Master Mix Set 1 was used according to the manufacturer's protocol (NEB). Briefly, 0.5 ug mRNA was used for fragmentation and then subjected to cDNA synthesis using SuperScript III Reverse Transcriptase (Invitrogen) and random primers. The cDNA was further converted into double stranded cDNA and after an end repair process (Klenow fragment, T4 polynucleotide kinase and T4 polymerase), was ligated to Illumina paired end (PE) adaptors. Size selection was performed using a 2% agarose gel, generating cDNA libraries ranging in size from 275–325 bp. Finally, the libraries were enriched using 15 cycles of PCR and purified by the QIAquick PCR purification kit (Qiagen).

### Sequence analysis

Libraries were sequenced on an Illumina GAIIx machine at the National Cancer Center, Singapore (Control-NCC, 16007-NCC, PDK13-NCC) or an Illumina HiSeq 2000 machine at the Duke-NUS Genome Biology Facility, Singapore (Control-1, Control-2, 16007-1, 16007-2, PDK13-1, PDK13-2). Resulting reads were mapped to the hg19 build of the human genome using Tophat v1.3.0 (http://tophat.cbcb.umd.edu/index.html) with the coverage-search, microexon-search and butterfly-search options. Differential isoform expression analysis was done using Cufflinks v1.3.0 (http://cufflinks.cbcb.umd.edu/) with the multi-read-correct (Cufflinks), -r and -s (Cuffcompare; using the same annotation gtf and hg19 fasta files respectively as in Tophat) and the mask-file (rRNA), frag-bias-correct (same hg19 fasta file used for Tophat) and multi-read-correct options. Differential splicing analysis was done using MISO v1.0 (http://genes.mit.edu/burgelab/miso/docs/) using the default options. Analysis of RNA sequencing quality was performed with RNAseqC v1.7 (http://www.broadinstitute.org/cancer/cga/rna-seqc) using the default options. Creation of proportional Venn diagrams was done with freeware available at www.venndiagram.tk. Hierarchical clustering of differentially regulated isoforms was done with Partek v6.6 (http://www.partek.com/). Pathway analysis of differentially regulated isoforms was done using Ingenuity Pathway Analysis v9.0 (http://www.ingenuity.com/).

### Validation of exon skipping by real-time PCR

Total RNA derived from mock-infected and DENV-infected cells was used to synthesize cDNA using SuperScript III First Strand Synthesis System (Invitrogen) with random hexamers according to manufacturer's instructions. Quantitative real-time PCR was performed using LightCycler 480 Real-Time PCR System (Roche Diagnostics GmbH, Germany) and LightCycler 480 SYBR Green I Master (Roche Diagnostics GmbH, Germany). The reaction was carried out to simultaneously amplify exon-skipped and exon-included isoforms using specific primers complementary to the exons flanking each target exon (Table S13). Percent exon exclusion levels were calculated as the percentage of the isoform excluding an alternative exon divided by the total abundance of the isoforms including and excluding the alternative exon.

### Statistical analysis

The statistical analysis employed to analyze biological triplicates for each condition are as previously described using the software Cufflinks and Mixture-of-Isoforms (MISO) [40], [41]. Criteria used to define significance are according to the standard options in the Cufflinks and MISO programs. More detail is provided below.

## Results

### Experimental design and RNAseq results

**Figure 1:**
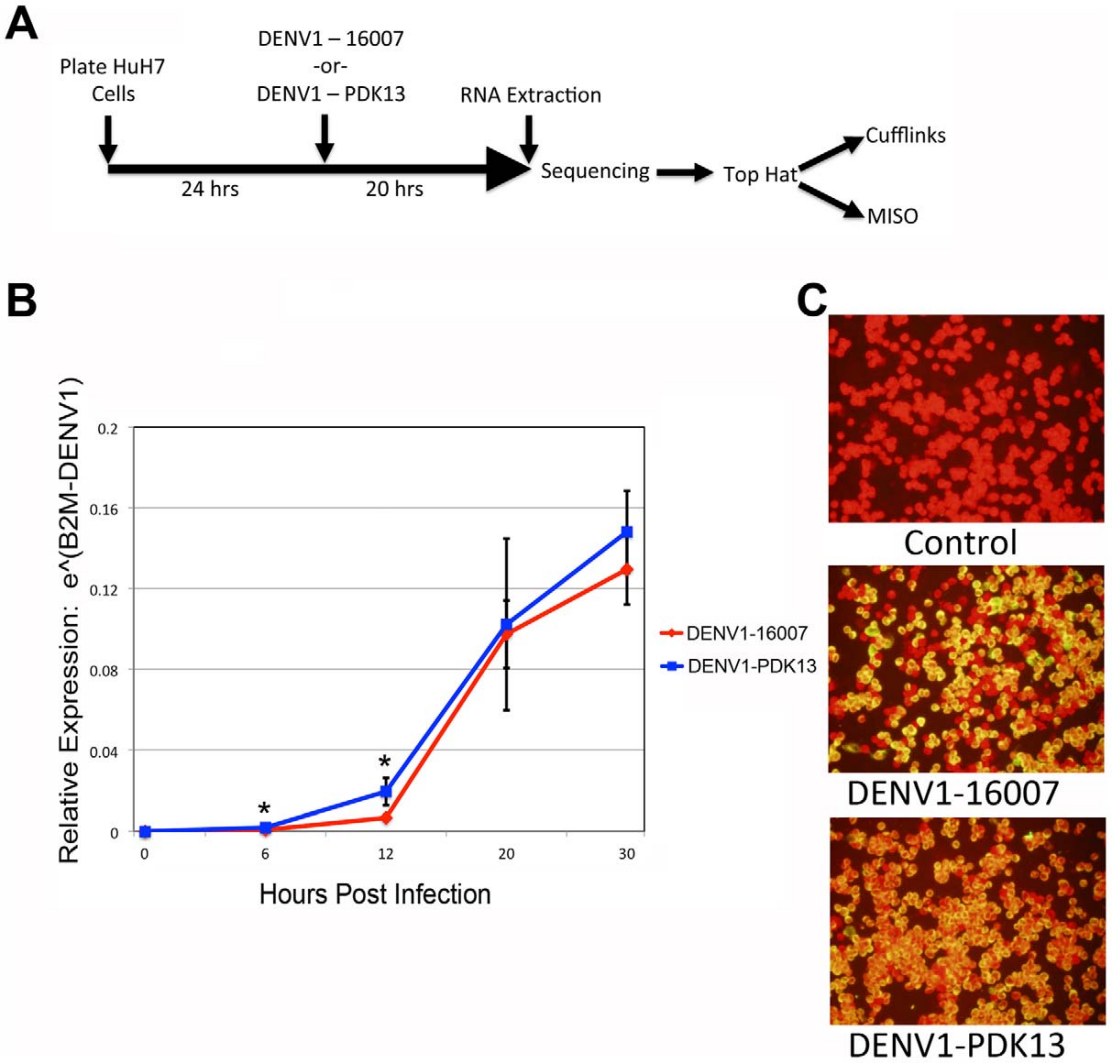
Experimental design and analysis of infection. **A**. Timeline of experimental procedure and data analysis steps. **B**. Quantification of viral copy number normalized to the cellular B2M gene expression at 0, 6, 12, 20 and 30 hours post infection. Asterisks indicate a p-value of less than or equal to 0.05. **C**. Immunofluorescence analysis of Huh7 cells either mock-infected (top panel), infected with DENV1-16007 (middle panel) or DENV1- PDK13 (bottom panel) and probed with antibody against prM protein (2H2) followed by FITC-conjugated goat anti-mouse IgG at 20 h post-infection. The cell nuclei were counter-stained with Evan's Blue.

To investigate the effect of DENV1-16007 (wild-type strain) and DENV1-PDK13 (attenuated strain) infection on the transcriptome of the human host, we infected human hepatoma cells (HuH7) with each strain for 20 hours at a multiplicity of infection (MOI) of 20 (Figure 1A). The MOI of 20 was chosen so that the subsequent RNAseq profiling would best reflect the infection-induced alterations to the host transcriptome and not be either masked by or derived from a large number of uninfected cells. We also measured viral RNA over the first 30 hours of infection to address the possibility that changes observed might be the result of a delayed replication cycle by one of the viruses. Although the absolute kinetics of the two viruses differ, the 20 hour time point was chosen as it represents the stage in the primary round of replication at which the genome copy numbers are most similar between the two viruses (Figure 1B). Indirect immunofluorescence staining for the pre-membrane protein (prM) production with 2H2 monoclonal antibody also showed similar infection levels for both viruses at this time point (Figure 1C). Infections with wild-type and attenuated strains were performed in three independent biological replicate experiments. Mock-infected HuH7 cells treated in the same way as the infected samples were also done in biological triplicate and served as the control for our experiment. At twenty hours post-infection, mRNA was extracted from all samples independently, and poly-A enriched cDNA libraries were constructed for 75-base, pair-end sequencing on an Illumina GAIIx (one sample for each condition) or Illumina HiSeq2000 machine (two samples for each condition). RNA sequencing was performed independently for each of these replicate experiments.

Mapping of reads (Bowtie, Tophat) and analysis of differential transcriptome response (Cufflinks) to infection was performed using the Tuxedo Suite of software [40], [42]. Cufflinks utilizes sequence fragments mapped to the reference genome to estimate the abundance of each isoform arising from the gene. It then tests for differential expression between experimental conditions. Differential expression at the gene level is calculated and is defined as the sum of differential expression of all isoforms at a particular locus. Cufflinks also assesses differential splicing by comparing the relative abundance of isoforms using the same transcriptional start site [40]. As this definition of splicing encompasses all different types of splicing events (see below) [43], systematic analysis of these events for downstream validation work is exceedingly difficult. In order to attain more detail about the types of differential splicing in our samples, we used the MISO software following mapping [41]. MISO utilizes a fixed library of previously characterized splice events to predict alternative splicing and reports the number of occurrences for each type of splicing event: skipped exon (SE), mutually exclusive exons (MXE), alternative 3′ splice site (A3SS), alternative 5′ splice site (A5SS), alternative first exon (AFE), retained intron (RI), tandem untranslated regions (Tandem UTR) [43]. MISO also utilizes a different algorithm than Cufflinks to predict differential splicing between samples which, when compared with the results from Cufflinks, provides an additional level of stringency in selection of candidates for downstream analysis.

To assess the quality of our libraries and sequencing performance and to ensure that any differences observed between our samples was due to the biology and not bias in sequencing, we used the open source program RNAseqC to examine our data [44]. Results indicate that despite using two different Illumina machines to generate the sequences, the individual samples are highly comparable to each other across all the metrics interrogated (Dataset S1).

### Wild-type and attenuated DENV-1 strains induce differential regulation of the host transcriptome

**Figure 2:**
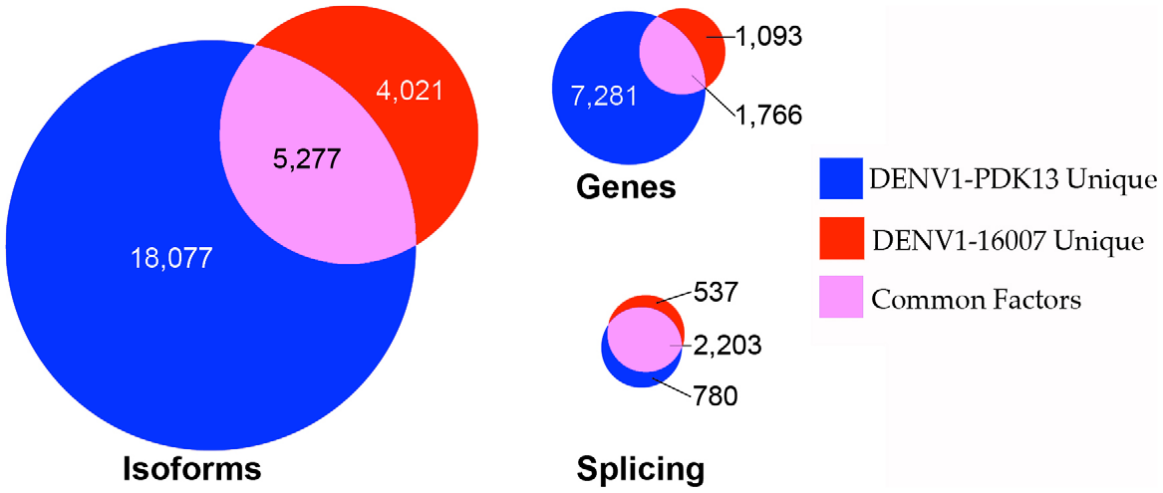
Comparison of differential regulation of genes, isoforms and splicing in DENV1-16007 and DENV1-PDK13.

Over 18,000 changes to the host transcriptome were observed in response to infection by the wild-type strain and >41,000 were observed in response to infection with the attenuated strain (Table 1). Differential isoform regulation is the largest category of response due to infection by both strains. Interestingly, there are over two-fold more differentially regulated isoforms following infection with the attenuated than with the wild-type strain. Similarly, infection with the attenuated strain also resulted in three-fold more differentially regulated genes than infection with the wild-type (Figure 2). In order to determine whether this large differential response to infection was specific to these two strains or whether the ‘quieter’ response to the parental DENV1-16007 strain was typical of wild-type DENV1s, we repeated our experiment in HuH7 cells with two low passaged clinical isolates (EDEN3300 and SL107) and compared them to our uninfected control. RNAseq analysis for these isolates indicates even fewer transcriptomic changes (Table 1), suggesting that attenuated virus triggers more host cell response than wild-type viruses. This observation is consistent with what has been reported for yellow fever virus and its attenuated derivative, YF17D [45].

**Table 1:** Differential regulation of transcripts in response to DENV1 infection.

Cufflinks	Uninfected^a^ vs PDK13^a^	Uninfected^a^ vs 16007^a^	Uninfected^a^ vs EDEN3300^b^	Uninfected^a^ vs SL107^b^
Genes exp	**9047** (4610 up/4437 down)	**2859** (1818 up/1041 down)	**5162** (109 up/5053 down)	**5033** (251 up/4782 down)
Isoforms exp	**23354** (12386 up/10968 down)	**9298** (5332 up/3966 down)	**6416** (775 up/5641 down)	**7023** (965 up/6058 down)
TSS group exp	**3189**	**1194**	**1429**	**1392**
CDS exp	**148**	**48**	**190**	**200**
CDS	**153**	**132**	**9**	**7**
Promoters	**2249**	**2112**	**148**	**184**
Splicing	**2983**	**2740**	**159**	**208**
*Total*	***41123***	***18383***	***13513***	***14047***

MISO	Uninfected^a^ vs PDK13^a^	Uninfected^a^ vs 16007^a^
SE	**515**	**322**
MXE	**76**	**55**
A3SS	**2**	**0**
A5SS	**2**	**5**
AFE	**0**	**0**
RI	**14**	**2**
Tandem UTR	**0**	**0**
*Total*	***609***	***384***

Number of events in each category (Rows) predicted by Cuffdiff [40] (Top) and MISO [41] (Bottom) for Uninfected cells vs. DENV1-16007 infected cells (First column), Uninfected cells vs. DENV1-PDK13 infected cells (Second column). The number of up- and down-regulated events is also described for the gene and isoform categories. Superscript “a” indicates biological triplicates while superscript “b” indicates single measurement of the sample. Abbreviations used: differentially expressed genes (Genes exp), differentially expressed isoforms (Isoforms exp), differentially expressed transcriptional start site group (TSS group exp), differentially expressed coding sequence (CDS exp), differential coding output (CDS), differential promoter use (Promoters), differential splicing (Splicing), skipped exon (SE), mutually exclusive exons (MXE), alternative 3′ splice site (A3SS), alternative 5′ splice site (A5SS), alternative first exon (AFE), retained intron (RI) and tandem untranslated region (Tandem UTR).

**Figure 3:**
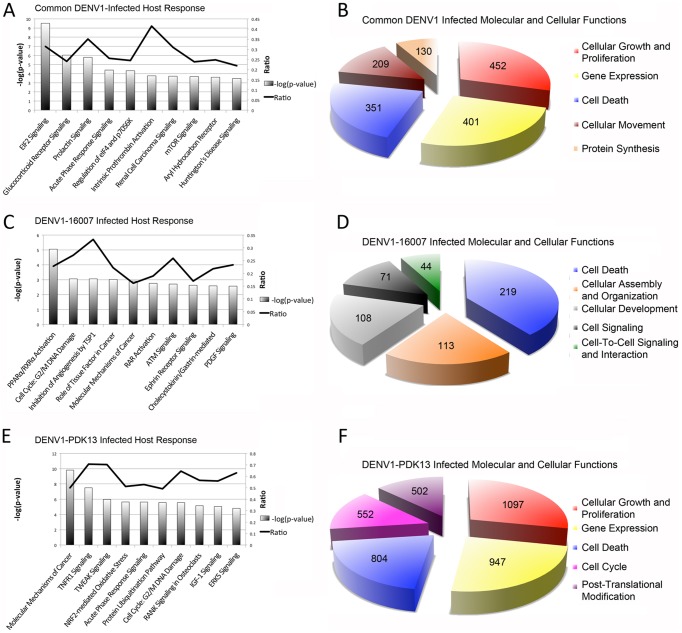
Ingenuity pathway analysis. Pathway (**A, C, E**) and molecular and cellular function (**B, D, F**) analysis of common DENV1 response (**A, B**), unique DENV1-16007 response (**C, D**) and unique DENV1-PDK13 response (**E, F**). The numbers within the pie-slices in panels B, D and F are the number of isoforms belonging to the indicated molecular and cellular function.

Qualitative analysis of the RNAseq data also provides insights into the pathways that are essential to both strains or unique to one strain. Ingenuity Pathway Analysis (IPA) of isoforms indicates that the commonly regulated isoforms are enriched in pathways associated with viral infection and modulation of protein translation. Examples include EIF2 signaling, prolactin signaling, acute phase response signaling, regulation of EIF4 and p7056K and glucocorticoid receptor signaling. Differential expression of pathways associated with cellular growth and proliferation such as mTOR signaling and aryl hydrocarbon receptor signaling are also enriched following infection with both strains (Figure 3A). Conversely, the pathways that are differentially regulated between the wild-type and attenuated strains include the innate immune response and cell cycle control. The top differentially regulated host pathways associated only with wild-type strain infection are involved with immunomodulation and cell cycle arrest, such as PPAR/RXR activation pathway, G2/M DNA damage checkpoint, ATM signaling and the PDGF signaling pathway (Figure 3C). Conversely, infection with the attenuated strain triggered pathways associated with inflammation, induction of apoptosis and stress such as the TNFR1 pathway, TWEAK signaling, NRF2-mediated oxidative stress response, IGF -1 signaling and ERK5 signaling (Figure 3E). IPA also identified molecular and cellular functions associated with both or each of the strains (Figures 3B, 3D and 3F). Taken collectively, the molecular and cellular functions in response to wild-type virus infection are associated with cell signaling and metabolism while those to attenuated virus are associated with transcriptional activation, cell cycle modification and post-translational modification.

### Differential splicing in the host transcriptome is largely conserved between wild-type and attenuated DENV1 strains

Next, we interrogated the data for alternative splicing within the isoforms sharing the same transcriptional start site. Interestingly, 80% and 74% of all differential splicing following infection with the wild-type strain and attenuated strain, respectively, are common to both viruses (Figure 2). This degree of commonality in the splicing response to infection by each strain is significantly different than what was observed for differential isoform and gene regulation in response to infection. This novel finding suggests that mechanisms responsible for the specific regulation of host splicing may be critical for DENV1 propagation and thus remained relatively unchanged during the attenuation process.

**Figure 4:**
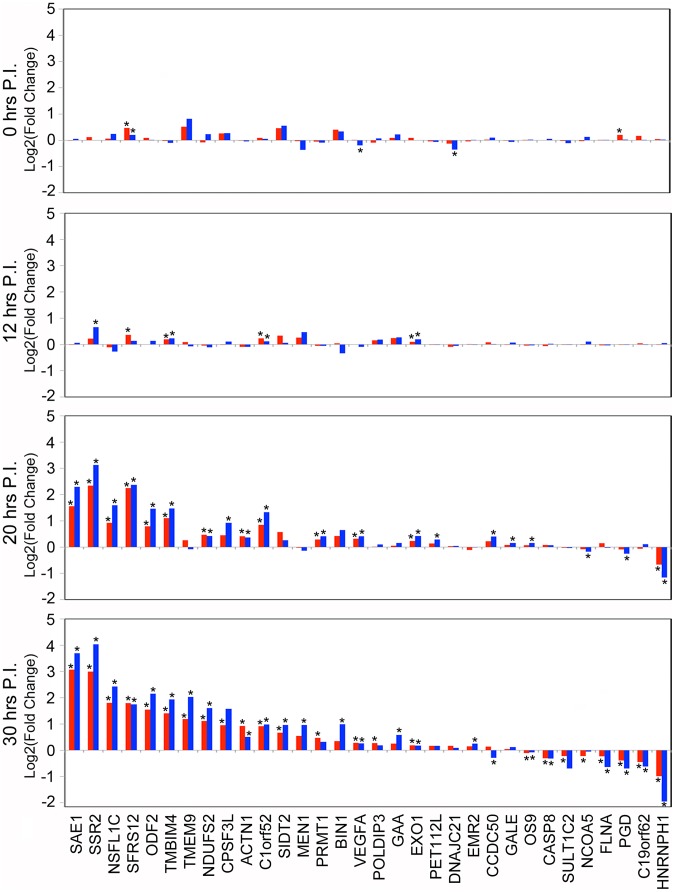
Validation of single exon skipping events predicted by both Cuffdiff and MISO. Values graphed are the log2 transformed fold change differences between inclusion/skipping of the alternatively spliced exon in DENV1-16007 infected cells (red bars) or DENV1-PDK13 infected cells (blue bars) compared to uninfected control cells for 0, 12, 20 and 30 hours post infection. Asterisks indicate a p-value of less than or equal to 0.05.

The candidate list from our Cufflinks analysis of alternatively spliced transcripts for both strains of virus was then cross-referenced against the list of skipped exon (SE) events generated by MISO analysis. This cross comparison resulted in 79 total SE events. To assess the accuracy of our alternative splicing predictions, we performed qPCR on each of the predicted SE events. Of the 79 events tested by qPCR, 32 (40%) were differentially expressed in the wild-type strain, the attenuated strain or both in comparison to an uninfected control (Figure 4). The splicing patterns were similar for both viruses across all time points measured, indicating that our observations are not artifacts of temporal sampling bias (Figure 4). Furthermore, the genes involved in these splicing events belong to many of the same pathways shown in Figure 3 suggesting that the virus is, at least in part, exerting its influence on these pathways through alternative splicing (Table S1). If this were true, the SE events should share mechanisms of regulation by utilizing common RNA motifs within and surrounding the identified SE's. Indeed, using the software RegRNA (http://regrna.mbc.nctu.edu.tw) [46] and setting an arbitrary boundary of 250 nucleotides upstream and downstream of the 3′ and 5′ splice-site of the SEs, respectively, we identified 74 predicted RNA regulatory motifs and elements which could be bound by 17 RNA binding proteins. By using GeneCards (http://www.genecards.org) to convert these putative motif binding proteins into HUGO nomenclature, we observed that nearly two thirds (11 of 17) of genes encoding these motif-binding proteins are themselves differentially expressed following infection by one or both of the DENV1 strains (Table S2). These results suggest that DENV1 may be regulating alternative splicing of host mRNA by a hitherto unknown mechanism.

### Cross-platform, integrative analysis of results

**Figure 5:**
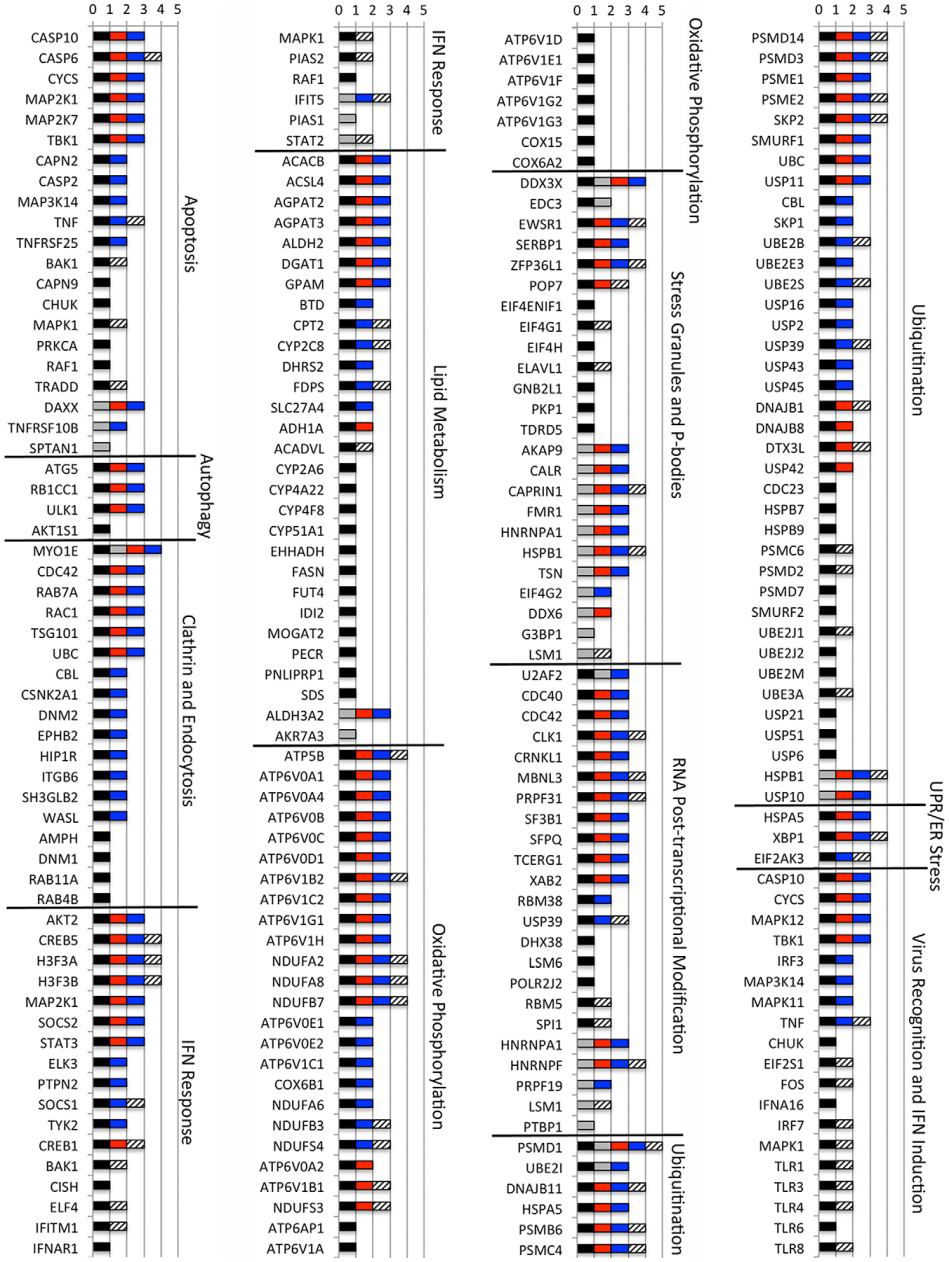
Cross-platform analysis of human-Flaviviridae interactions. Members of eleven canonical pathways that have been previously implicated to be important for DENV propagation were interrogated for their appearance in an RNAi screen for Flaviviridae host factors, direct interaction with DENV gene products, differential regulation in a microarray study, differential isoform regulation following infection with DENV1-16007 and/or differential isoform regulation following infection with DENV1-PDK13. Host factors identified in published and/or unpublished Flaviviridae RNAi screens [10], [11], [48], [49], [51] (Jamison and Garcia-Blanco, unpublished data) were combined and are indicated by a black bar. Host proteins found to interact DENV gene products [47], [50] were combined and are indicated by a grey bar. Host genes found to be differentially regulated in microarray studies [12]–[29] are indicated by the striped bar. A red bar indicates host isoforms found to be differentially regulated during infection with DENV1-16007. A blue bar indicates host isoforms found to be differentially regulated during infection with DENV1-PDK13.

Many differentially processed transcripts found in our study have also been identified in genome-wide RNAi screens for flavivirus host factors, differentially processed in microarray studies of DENV-infected cells and/or shown to interact with DENV gene products [10]–[29], [47]–[51] (Jamison and Garcia-Blanco, unpublished data). To gain additional functional insights into the differentially regulated transcripts identified here, we compiled a cross-platform integrative analysis of our data with other available genomic data on host factors in DENV and other flaviviral infection (Tables S3, S4, S5, S6, S7, S8, S9, S10, S11, S12, S13). Briefly, we examined 11 canonical pathways that were enriched in our study and/or had been previously implicated in the DENV life cycle: apoptosis, autophagy, clathrin-mediated endocytosis, interferon signaling, lipid metabolism, oxidative phosphorylation, regulation of stress granules and P-bodies, splicing-related RNA post-transcriptional modification, ubiquitination, endoplasmic reticulum stress, virus recognition and interferon induction. The proportion of host factors for DENV or other flaviviruses identified through functional genomic studies that were differentially regulated following infection with the wild-type strain or the attenuated strain is indicated in Table 2. Differential isoform regulation of flaviviral host factors ranged from 15.5% to 60.6% and is uniformly higher for the attenuated strain than the pathogenic wild-type strain. Alternative splicing ranged from 7.3% to 24.2% and the rates of these events in the wild-type strain and its attenuated derivative were comparable (Table 2). The intersection of specific host factors in these eleven canonical pathways that are either required for flaviviral propagation or involved in direct interaction with DENV proteins, which are differentially regulated are graphically depicted in Figure 5.

**Table 2:** Cross-platform analysis of DENV host factors.

Screen	Reference	Cell Line Used	Identified	Isoform	Isoform	Splicing	Splicing
DENV (Drosophila)	Sessions *et al* 2009	S2, HuH7	56	14 (25%)	24 (42.9%)	7 (12.5%)	7 (12.5%)
DENV (Human)	Jamison *et al* Unpublished	HuH7	943	191 (20.3%)	360 (38.2%)	90 (9.5%)	89 (9.4%)
HCV-Replicon	Tai *et al* 2009	HuH7/Rep-Feo	97	27 (27.8%)	43 (44.3%)	12 (12.4%)	12 (12.4%)
HCV-Virus	Li *et al* 2009	HuH7.5.1	263	68 (25.9%)	115 (43.7%)	30 (11.4%)	29 (11%)
WNV	Krishnan *et al* 2008	HeLa	306	54 (17.6%)	105 (34.3%)	29 (9.5%)	34 (11.1%)
WNV	Krishnan *et al* 2008 (Unpublished Hits)	HeLa	1259	195 (15.5%)	369 (29.3%)	92 (7.3%)	101 (8%)
YFV	Le Sommer *et al* 2012	HuH7	647	116 (17.9%)	217 (33.5%)	49 (7.6%)	54 (8.3%)
DENV UTR-BP's	Ward *et al* 2011	HuH7	62	26 (41.9%)	34 (54.8%)	14 (22.6%)	15 (24.2%)
DENV-Y2H	Le Breton *et al* 2011	*S. cerevisae*, HEK293T	104	45 (43.3%)	63 (60.6%)	16 (15.4%)	17 (16.3%)

The number of host factors identified in each of the RNAi screens listed compared to the differentially regulated isoforms and differentially spliced genes for both the wild-type DENV1-16007 and vaccine strain DENV1-PDK13. Percentages of overlap are indicated in parenthesis. The unpublished DENV screen by Jamison SF, Garcia-Blanco, M.A. utilize the same siRNA library, cell line, general methodology and statistical analysis described in Le Sommer et al 2012. The unpublished hits from the WNV screen are the full, unannotated results from the published Krishnan et al 2008 study.

## Discussion

Investigation of host gene expression to date has relied primarily on microarray technology [12]–[29]. This technology is insensitive to the regulation of genes through alternative RNA processing. Thus, detection of differential expression down to the level of alternative isoforms has not been examined and the subtleties of TSS and post-transcriptional modifications that can dramatically alter the function of the derived proteins have been largely ignored. These processes could be a mechanism by which DENV attains specific isoforms of required host factors while suppressing those that act to restrict its replication [27], [52], [53]. Indeed, the need to understand the host response beyond simple gene expression is underscored by the observation that DENV can modify the splicing pattern of an endogenous gene, XBP1, to its advantage [53]. Our findings indicate that there is an extensive amount of previously uncharacterized gene isoforms and alternative processing of host transcripts over a wide range of pathways and host functions in response to DENV infection. Interestingly, the DENV1-16007 and DENV1-PDK13 viruses only differ from each other by 14 nucleotide and 8 amino acids [54], yet the host transcriptional response to these viruses is pronounced. This suggests that infection with different strains of DENV can result in significantly different disease phenotypes despite few nucleotide differences.

We have attempted, in this study, to provide a comprehensive guide to the transcriptomic changes with DENV infection. By analyzing our RNAseq data using two different programs, Cufflinks and MISO [40], [41], maximal information on RNA transcript regulation, could be gleaned. Specific splice events could hence be identified for subsequent mechanistic studies that clarify their role in the host response. In particular, the integrative analysis of this study with existing functional genomics data reveals previously undocumented expression and post-transcriptional regulation of required host factors that should serve as a road map for future mechanistic investigations. A caveat, however, is that our work only profiled the transcriptome at the end of a single round of DENV replication. As hinted by Figure 4, both quantitative and qualitative differences may exist in the transcriptome at different stages of the virus life cycle. Furthermore, the possibility exists that bystander uninfected cells may exert some influence on the observed transcriptional changes although we have attempted to minimize this by using a high MOI in our experiments. Future studies may need to take these possibilities into account.

The large difference in the number of alternative splicing events identified by Cufflinks and MISO also underscores the fledgling nature of RNAseq. While the former identifies all possible splicing events from the data *de novo*, the latter relies on a pre-defined library to map alternatively spliced transcripts. Additional studies are needed to clarify whether Cufflinks over-estimated the number of splice variants, or there are authentic variants absent from the library used by MISO. Regardless, for transcriptome analysis of host response to infection, RNAseq is superior to microarray in terms of the breadth of information derived.

Our results also indicate that the human transcriptome response to an infection by wild-type DENV or its attenuated derivative differs significantly (Table 1, Figure 2). These differences suggest that alternative processing events may be part of a previously uncharacterized innate immune response to DENV1 infection that is in large part evaded by wild-type strains. This second hypothesis is supported by the greater than two-fold increase in the number of differentially regulated transcripts when infected with the attenuated strain of DENV1 as compared to the parental wild type strain of DENV1, many of which belong to pathways associated with inflammation, induction of apoptosis and stress response (Table 1, Figure 3). This inability to escape the innate immune response achieved by the wild-type virus may explain the lack of reactogenicity. It may also explain why antibody titer engendered by vaccination is not as high as those observed following natural infection unless supplemented with an adjuvant that stimulates this innate immune response. This observation also suggests a mechanism of pathogenicity where DENV regulates host transcriptome changes by interacting with a group of RNA binding proteins to control multiple splicing events.

The development of a live attenuated tetravalent vaccine for DENV1-4 has bedeviled researchers for the past 60 years. The less than optimal efficacy of the leading dengue vaccine candidate makes an improved understanding of the molecular basis of a good vaccine all the more critical [55]. The differential host transcriptome response to infection with DENV1-16007 and DENV1-PDK13 provides an insight into the characteristics of an attenuated virus which, is likely a complex phenotype [45]. A molecular understanding of the basis of attenuation could lead to a quantitative approach to balancing reactogenicity and immunogenicity, which presently remains a hit-or-miss finding made only after lengthy clinical trials.

In conclusion, we provide here a detailed view of the host cell transcriptome response to infection with wild-type DENV-1 and its attenuated derivative that could be useful for future studies on the genetic determinants of viral virulence and attenuation.

## Supporting Information

Dataset S1 **RNASeQC analysis.** To assess the quality of our sequencing reactions and mapping, we used the open-source software RNASeQC available at: http://www.broadinstitute.org/cancer/cga/rna-seqc. Information about Total Reads, Mapped Reads, Mate Pairs, Transcript-associated Reads and Strand Specificity are output. Details about each information type are listed below the corresponding table. Additionally, a Spearman Correlation Matrix and Pearson Correlation Matrix were produced for the samples. Tables listing the Coverage Metrics are produced for the Bottom 1000 Expressed Transcripts, the Middle 1000 Expressed Transcripts and the Top 1000 Expressed Transcripts. Graphs depicting the Mean Coverage over the Percentage of Transcript Length (5′ to 3′) and graphs depicting Mean Coverage from the 3′ End for Low Expressed Transcripts, Medium Expressed Transcripts and High Expressed Transcripts are also provided.
(PDF)

Table S1 **Ingenuity pathway analysis of the 33 skipped exon events found to be differentially regulated by qPCR.** −log(p-value) reflects the statistical significance of the indicated observation while the ratio refers to the number of genes identified in the indicated pathway divided by the total number of genes belonging to that pathway.
(XLS)

Table S2 **Motif analysis of the RNA surrounding skipped exons.** The 500 base areas surrounding the skipped exon described in Figure 4, as well as the exons themselves were interrogated for the presence of RNA regulatory motifs and elements. The predicted motif binding proteins were translated into HUGO nomenclature and checked for differential regulation following infection with DENV1-16007, DENV1-PDK13 or both.
(XLS)

Table S3 **Cross-platform analysis of the apoptosis pathway.** Members of the apoptosis pathway were interrogated for their appearance in an RNAi screen for *Flaviviridae* host factors [10], [11], [48], [49], [51] (Jamison and Garcia-Blanco, unpublished data), direct interaction with DENV gene products [47], [50] and differential regulation in a microarray study [12]–[29]. Differential regulation following infection with DENV1-16007 and/or DENV1-PDK13 was also interrogated for each member of these pathways with the Cuffdiff values from the current experiment recorded in each of the corresponding columns.
(XLS)

Table S4 **Cross-platform analysis of the autophagy pathway.** Members of the autophagy pathway were interrogated for their appearance in an RNAi screen for *Flaviviridae* host factors [10], [11], [48], [49], [51] (Jamison and Garcia-Blanco, unpublished data), direct interaction with DENV gene products [47], [50] and differential regulation in a microarray study [12]–[29]. Differential regulation following infection with DENV1-16007 and/or DENV1-PDK13 was also interrogated for each member of these pathways with the Cuffdiff values from the current experiment recorded in each of the corresponding columns.
(XLS)

Table S5 **Cross-platform analysis of the clathrin-mediated endocytosis pathway.** Members of the clathrin-mediated endocytosis pathway were interrogated for their appearance in an RNAi screen for *Flaviviridae* host factors [10], [11], [48], [49], [51] (Jamison and Garcia-Blanco, unpublished data), direct interaction with DENV gene products [47], [50] and differential regulation in a microarray study [12]–[29]. Differential regulation following infection with DENV1-16007 and/or DENV1-PDK13 was also interrogated for each member of these pathways with the Cuffdiff values from the current experiment recorded in each of the corresponding columns.
(XLS)

Table S6 **Cross-platform analysis of the interferon signaling pathway.** Members of the interferon signaling pathway were interrogated for their appearance in an RNAi screen for *Flaviviridae* host factors [10], [11], [48], [49], [51] (Jamison and Garcia-Blanco, unpublished data), direct interaction with DENV gene products [47], [50] and differential regulation in a microarray study [12]–[29]. Differential regulation following infection with DENV1-16007 and/or DENV1-PDK13 was also interrogated for each member of these pathways with the Cuffdiff values from the current experiment recorded in each of the corresponding columns.
(XLS)

Table S7 **Cross-platform analysis of the lipid metabolism pathway.** Members of the lipid metabolism pathway were interrogated for their appearance in an RNAi screen for *Flaviviridae* host factors [10], [11], [48], [49], [51] (Jamison and Garcia-Blanco, unpublished data), direct interaction with DENV gene products [47], [50] and differential regulation in a microarray study [12]–[29]. Differential regulation following infection with DENV1-16007 and/or DENV1-PDK13 was also interrogated for each member of these pathways with the Cuffdiff values from the current experiment recorded in each of the corresponding columns.
(XLS)

Table S8 **Cross-platform analysis of the oxidative phosphorylation pathway.** Members of the oxidative phosphorylation pathway were interrogated for their appearance in an RNAi screen for *Flaviviridae* host factors [10], [11], [48], [49], [51] (Jamison and Garcia-Blanco, unpublished data), direct interaction with DENV gene products [47], [50] and differential regulation in a microarray study [12]–[29]. Differential regulation following infection with DENV1-16007 and/or DENV1-PDK13 was also interrogated for each member of these pathways with the Cuffdiff values from the current experiment recorded in each of the corresponding columns.
(XLS)

Table S9 **Cross-platform analysis of the regulation of stress granules and p-bodies pathway.** Members of the regulation of stress granules and p-bodies pathway were interrogated for their appearance in an RNAi screen for *Flaviviridae* host factors [10], [11], [48], [49], [51] (Jamison and Garcia-Blanco, unpublished data), direct interaction with DENV gene products [47], [50] and differential regulation in a microarray study [12]–[29]. Differential regulation following infection with DENV1-16007 and/or DENV1-PDK13 was also interrogated for each member of these pathways with the Cuffdiff values from the current experiment recorded in each of the corresponding columns.
(XLS)

Table S10 **Cross-platform analysis of the splicing related RNA post-transcriptional modification pathway.** Members of the splicing related RNA post-transcriptional modification pathway were interrogated for their appearance in an RNAi screen for *Flaviviridae* host factors [10], [11], [48], [49], [51] (Jamison and Garcia-Blanco, unpublished data), direct interaction with DENV gene products [47], [50] and differential regulation in a microarray study [12]–[29]. Differential regulation following infection with DENV1-16007 and/or DENV1-PDK13 was also interrogated for each member of these pathways with the Cuffdiff values from the current experiment recorded in each of the corresponding columns.
(XLS)

Table S11 **Cross-platform analysis of the ubiquitination pathway.** Members of the ubiquitination pathway were interrogated for their appearance in an RNAi screen for *Flaviviridae* host factors [10], [11], [48], [49], [51] (Jamison and Garcia-Blanco, unpublished data), direct interaction with DENV gene products [47], [50] and differential regulation in a microarray study [12]–[29]. Differential regulation following infection with DENV1-16007 and/or DENV1-PDK13 was also interrogated for each member of these pathways with the Cuffdiff values from the current experiment recorded in each of the corresponding columns.
(XLS)

Table S12 **Cross-platform analysis of the endoplasmic reticulum stress pathway.** Members of the endoplasmic reticulum stress pathway were interrogated for their appearance in an RNAi screen for *Flaviviridae* host factors [10], [11], [48], [49], [51] (Jamison and Garcia-Blanco, unpublished data), direct interaction with DENV gene products [47], [50] and differential regulation in a microarray study [12]–[29]. Differential regulation following infection with DENV1-16007 and/or DENV1-PDK13 was also interrogated for each member of these pathways with the Cuffdiff values from the current experiment recorded in each of the corresponding columns.
(XLS)

Table S13 **Cross-platform analysis of the virus recognition and interferon induction pathway.** Members of the virus recognition and interferon induction pathway were interrogated for their appearance in an RNAi screen for *Flaviviridae* host factors [10], [11], [48], [49], [51] (Jamison and Garcia-Blanco, unpublished data), direct interaction with DENV gene products [47], [50] and differential regulation in a microarray study [12]–[29]. Differential regulation following infection with DENV1-16007 and/or DENV1-PDK13 was also interrogated for each member of these pathways with the Cuffdiff values from the current experiment recorded in each of the corresponding columns.
(XLS)

Table S14 **Primers.** The primer sequences used to validate the SE exon events are detailed here.
(XLS)
