# Supplementary material for: Host Cell Transcriptome Profile during Wild-Type and Attenuated Dengue Virus Infection
Source: PLoS Negl Trop Dis. 2013 Mar 14;7(3):e2107. doi: 10.1371/journal.pntd.0002107 (PMC3597485; doi:10.1371/journal.pntd.0002107)
Supplement: Dataset S1 — RNASeQC analysis. To assess the quality of our sequencing reactions and mapping, we used the open-source software RNASeQC available at: http://www.broadinstitute.org/cancer/cga/rna-seqc. Information about Total Reads, Mapped Reads, Mate Pairs, Transcript-associated Reads and Strand Specificity are output. Details about each information type are listed below the corresponding table. Additionally, a Spearman Correlation Matrix and Pearson Correlation Matrix were produced for the samples. Tables listing the Coverage Metrics are produced for the Bottom 1000 Expressed Transcripts, the Middle 1000 Expressed Transcripts and the Top 1000 Expressed Transcripts. Graphs depicting the Mean Coverage over the Percentage of Transcript Length (5′ to 3′) and graphs depicting Mean Coverage from the 3′ End for Low Expressed Transcripts, Medium Expressed Transcripts and High Expressed Transcripts are also provided. (PDF) [file pntd.0002107.s001.pdf]

RNA-seq Metrics

Read Count Metrics

The following summary statistics are calculated by counting the number of reads that have the given characteristics.

Total Reads

Sample	Note	Total Purity Filtered Reads Sequenced	Alternative Aligments	Failed Vendor QC Check	Read Length	Estimated Library Size
Control-NCC	11May2012	43,428,578	2,282,793	NA	79	NA
Control-1	11May2012	203,062,932	13,384,040	NA	74	NA
Control-2	11May2012	201,509,835	10,440,862	NA	74	NA
16007-NCC	11May2012	49,505,435	2,686,125	NA	79	NA
16007-1	11May2012	177,008,876	9,443,504	NA	74	NA
16007-2	11May2012	194,203,549	9,434,638	NA	74	NA
PDK13-NCC	11May2012	46,266,956	2,146,382	NA	79	NA
PDK13-1	11May2012	168,335,185	6,907,404	NA	74	NA
PDK13-2	11May2012	194,423,341	8,691,098	NA	74	NA

Total Purity Filtered Reads Sequenced are filtered for vendor fail flags and exclude alternative alignment reads. **Alternative Aligments** are duplicate read entries providing alternative coordinates. **Failed Vendor QC Check** are reads which have been designated as failed by the sequencer. **Read Length** is the maximum length found for all reads. **Estimated Library Size** is the number of expected fragments based upon the total number of reads and duplication rate assuming a Poisson distribution.

Mapped Reads

Sample	Note	Mapped	Mapping Rate	Mapped Unique	Mapped Unique Rate of Total	Unique Rate of Mapped	Duplication Rate of Mapped	Base Mismatch Rate	rRNA	rRNA rate
Control-NCC	11May2012	43,428,578	1.000	43,428,578	1.000	1.000	NA	0.008	NA	NA
Control-1	11May2012	203,062,932	1.000	203,062,932	1.000	1.000	NA	0.007	NA	NA
Control-2	11May2012	201,509,835	1.000	201,509,835	1.000	1.000	NA	0.007	NA	NA
16007-NCC	11May2012	49,505,435	1.000	49,505,435	1.000	1.000	NA	0.005	NA	NA
16007-1	11May2012	177,008,876	1.000	177,008,876	1.000	1.000	NA	0.008	NA	NA
16007-2	11May2012	194,203,549	1.000	194,203,549	1.000	1.000	NA	0.006	NA	NA
PDK13-NCC	11May2012	46,266,956	1.000	46,266,956	1.000	1.000	NA	0.005	NA	NA
PDK13-1	11May2012	168,335,185	1.000	168,335,185	1.000	1.000	NA	0.006	NA	NA
PDK13-2	11May2012	194,423,341	1.000	194,423,341	1.000	1.000	NA	0.006	NA	NA

Mapped reads are those that were aligned. **Mapping Rate** is per total reads. **Mapped Unique** are both aligned as well as

non-duplicate reads. **Mapped Unique Rate of Total** is per total reads. **Unique Rate of Mapped** are unique reads divided by all mapped reads. **Duplication Rate of Mapped** is the duplicate read divided by total mapped reads. **Base Mismatch Rate** is the number of bases not matching the reference divided by the total number of aligned bases. **rRNA** reads are non-duplicate and duplicate reads aligning to rRNA regions as defined in the transcript model definition. **rRNA Rate** is per total reads.

Mate Pairs

Sample	Note	Mapped Pairs	Unpaired Reads	End 1 Mapping Rate	End 2 Mapping Rate	End 1 Mismatch Rate	End 2 Mismatch Rate	Fragment Length Mean	Fragment Length StdDev	Chimeric Pairs
Control-NCC	11May2012	19,835,158	NA	1.017	0.983	0.008	0.008	130	117	330,988
Control-1	11May2012	92,669,320	NA	1.014	0.986	0.007	0.007	286	293	1,583,189
Control-2	11May2012	90,116,306	NA	1.018	0.982	0.007	0.007	285	290	1,375,358
16007-NCC	11May2012	22,802,757	NA	1.010	0.990	0.005	0.005	125	118	405,403
16007-1	11May2012	78,177,347	NA	1.026	0.974	0.008	0.009	277	283	1,055,545
16007-2	11May2012	87,986,654	NA	1.014	0.986	0.006	0.007	292	286	1,163,151
PDK13-NCC	11May2012	21,303,160	NA	1.009	0.991	0.004	0.005	126	116	289,050
PDK13-1	11May2012	77,122,597	NA	1.009	0.991	0.005	0.006	298	283	833,538
PDK13-2	11May2012	88,350,197	NA	1.014	0.986	0.006	0.006	304	287	1,040,304

Mapped Pairs is the total number of pairs for which both ends map. **Unpaired Reads** are the number of reads that are lacking a mate. **End 1/2 Mapping Rate** is the number of mapped divided by the total number of End1/End2 reads. **End 1/2 Mismatch Rate** is the number of End 1 and 2 bases not matching the reference divided by the total number of mapped End 1 and 2 bases. **Fragment Length Mean/StdDev** is the mean distance, standard deviation between the start of an upstream read and the end of the downstream one. Only fragments contained within single exons are used. **Chimeric Pairs** are pairs whose mates map to different genes.

Transcript-associated Reads

Sample	Note	Intragenic Rate	Exonic Rate	Intronic Rate	Intergenic Rate	Expression Profiling Efficiency	Transcripts Detected	Genes Detected
Control-NCC	11May2012	0.975	0.877	0.099	0.024	0.877	91,003	20,318
Control-1	11May2012	0.973	0.873	0.100	0.026	0.873	101,120	24,366
Control-2	11May2012	0.973	0.871	0.102	0.026	0.871	102,545	24,896
16007-NCC	11May2012	0.961	0.807	0.154	0.039	0.807	94,167	21,755
16007-1	11May2012	0.970	0.859	0.111	0.030	0.859	102,653	25,067
16007-2	11May2012	0.970	0.860	0.110	0.029	0.860	103,127	25,184
PDK13-NCC	11May2012	0.966	0.830	0.136	0.034	0.830	93,073	21,240
PDK13-1	11May2012	0.973	0.868	0.105	0.027	0.868	101,917	24,447
PDK13-2	11May2012	0.971	0.865	0.106	0.029	0.865	103,115	25,121

All of the above rates are per mapped read. **Intragenic Rate** refers to the fraction of reads that map within genes (within introns or exons). **Exonic Rate** is the fraction mapping within exons. **Intronic Rate** is the fraction mapping within introns. **Intergenic Rate** is the fraction mapping in the genomic space between genes. **Expression Profile Efficiency** is the ratio of

exon reads to total reads. **Transcripts/Genes Detected** is the number of transcripts/Genes with at least 5 reads.

Strand Specificity

Sample	Note	End 1 Sense	End 1 Antisense	End 2 Sense	End 2 Antisense	End 1 % Sense	End 2 % Sense
Control-NCC	11May2012	9,341,434	9,687,271	9,184,603	9,204,740	49.091	49.945
Control-1	11May2012	43,906,705	44,562,293	42,565,561	43,362,294	49.629	49.536
Control-2	11May2012	43,785,825	45,045,034	42,534,315	43,075,409	49.291	49.684
16007-NCC	11May2012	10,401,221	10,852,632	10,313,746	10,528,029	48.938	49.486
16007-1	11May2012	38,922,976	39,768,493	37,115,313	37,507,323	49.463	49.737
16007-2	11May2012	41,963,715	43,231,407	41,405,368	41,476,743	49.256	49.957
PDK13-NCC	11May2012	9,818,472	10,217,006	9,766,692	9,906,265	49.005	49.645
PDK13-1	11May2012	36,496,356	37,299,198	36,239,050	36,202,751	49.456	50.025
PDK13-2	11May2012	42,298,318	43,198,275	41,354,005	41,798,849	49.474	49.733

End 1/2 Sense are the number of End 1 or 2 reads that were sequenced in the sense direction. Similarly, **End 1/2 Antisense** are the number of End 1 or 2 reads that were sequenced in the antisense direction.**End 1/2 Sense %** are percentages of intragenic End 1/2 reads that were sequenced in the sense direction.

Correlation Analysis

Spearman Correlation Matrix

Sample	Note	Control-NCC	Control-1	Control-2	16007-NCC	16007-1	16007-2	PDK13-NCC	PDK13-1	PDK13-2
Control-NCC	11May2012	1.000	0.984	0.978	0.981	0.974	0.975	0.970	0.962	0.960
Control-1	11May2012	0.984	1.000	0.982	0.984	0.981	0.980	0.971	0.965	0.966
Control-2	11May2012	0.978	0.982	1.000	0.978	0.986	0.988	0.968	0.974	0.972
16007-NCC	11May2012	0.981	0.984	0.978	1.000	0.978	0.980	0.977	0.967	0.967
16007-1	11May2012	0.974	0.981	0.986	0.978	1.000	0.990	0.976	0.982	0.985
16007-2	11May2012	0.975	0.980	0.988	0.980	0.990	1.000	0.977	0.983	0.984
PDK13-NCC	11May2012	0.970	0.971	0.968	0.977	0.976	0.977	1.000	0.980	0.980
PDK13-1	11May2012	0.962	0.965	0.974	0.967	0.982	0.983	0.980	1.000	0.990
PDK13-2	11May2012	0.960	0.966	0.972	0.967	0.985	0.984	0.980	0.990	1.000

Pearson Correlation Matrix

Sample	Note	Control-NCC	Control-1	Control-2	16007-NCC	16007-1	16007-2	PDK13-NCC	PDK13-1	PDK13-2
Control-NCC	11May2012	1.000	0.988	0.981	0.981	0.978	0.979	0.972	0.967	0.966
Control-1	11May2012	0.988	1.000	0.986	0.986	0.985	0.984	0.975	0.971	0.972
Control-2	11May2012	0.981	0.986	1.000	0.980	0.990	0.992	0.972	0.980	0.979
16007-NCC	11May2012	0.981	0.986	0.980	1.000	0.981	0.982	0.978	0.970	0.971
16007-1	11May2012	0.978	0.985	0.990	0.981	1.000	0.994	0.979	0.987	0.989
16007-2	11May2012	0.979	0.984	0.992	0.982	0.994	1.000	0.980	0.988	0.988

PDK13-NCC	11May2012	0.972	0.975	0.972	0.978	0.979	0.980	1.000	0.982	0.982
PDK13-1	11May2012	0.967	0.971	0.980	0.970	0.987	0.988	0.982	1.000	0.994
PDK13-2	11May2012	0.966	0.972	0.979	0.971	0.989	0.988	0.982	0.994	1.000

Coverage Metrics for Bottom 1000 Expressed Transcripts

The metrics in this table are calculated across the transcripts that were determined to have the highest expression levels.

Sample	Note	Mean Per Base Cov.	Mean CV	No. Covered 5'	5'200Base Norm	No. Covered 3'	3' 200Base Norm	Num. Gaps	Cumul. Gap Length	Gap %
Control-NCC	11May2012	4.10	1.23	639	0.66	494	5371	596330	29.4	
Control-1	11May2012	17.19	1.16	797	0.60	717	2967	237245	12.6	
Control-2	11May2012	17.29	1.10	795	0.69	736	2680	308114	14.3	
16007-NCC	11May2012	4.49	1.11	670	0.58	557	4710	452755	24.1	
16007-1	11May2012	15.11	1.09	755	0.49	724	3016	257142	13.0	
16007-2	11May2012	16.60	1.08	798	0.67	750	2950	256240	13.1	
PDK13-NCC	11May2012	4.19	1.09	627	0.62	550	4664	470199	24.4	
PDK13-1	11May2012	14.35	1.10	785	0.80	716	2998	276035	15.2	
PDK13-2	11May2012	16.74	1.04	779	0.53	775	2440	192543	10.8	

It is important to note that these values are restricted to the bottom 1000 expressed transcripts. **5'** and **3'** values are per-base coverage averaged across all top transcripts. 5' and 3' ends are 200 base pairs. Gap % is the total cumulative gap length divided by the total cumulative transcript lengths.

Coverage Metrics for Middle 1000 Expressed Transcripts

The metrics in this table are calculated across the transcripts that were determined to have the highest expression levels.

Sample	Note	Mean Per Base Cov.	Mean CV	No. Covered 5'	5'200Base Norm	No. Covered 3'	3' 200Base Norm	Num. Gaps	Cumul. Gap Length	Gap %
Control-NCC	11May2012	29.80	0.94	828	0.45	767	2098	172239	8.2	
Control-1	11May2012	128.27	0.96	900	0.42	900	1054	70326	3.7	
Control-2	11May2012	128.67	0.89	911	0.42	902	966	72575	3.9	
16007-NCC	11May2012	30.97	0.88	835	0.40	836	1801	133199	6.4	
16007-1	11May2012	116.38	0.90	887	0.40	917	1042	68918	3.6	
16007-2	11May2012	126.81	0.85	898	0.39	921	995	66709	3.4	
PDK13-NCC	11May2012	31.53	0.88	808	0.37	811	2040	150957	7.2	
PDK13-1	11May2012	120.82	0.89	899	0.42	895	1094	79204	4.0	
PDK13-	11May2012	134.90	0.87	901	0.37	931	944	58402	2.9	

2										

It is important to note that these values are restricted to the middle 1000 expressed transcripts. **5'** and **3'** values are per-base coverage averaged across all top transcripts. 5' and 3' ends are 200 base pairs. Gap % is the total cumulative gap length divided by the total cumulative transcript lengths.

Coverage Metrics for Top 1000 Expressed Transcripts

The metrics in this table are calculated across the transcripts that were determined to have the highest expression levels.

Sample	Note	Mean Per Base Cov.	Mean CV	No. Covered 5'	5'200Base Norm	No. Covered 3'	3' 200Base Norm	Num. Gaps	Cumul. Gap Length	Gap %
Control-NCC	11May2012	-255.12	0.83	923	0.29	960	488	38655	3.2	
Control-1	11May2012	238.83	0.86	950	0.28	991	234	21126	1.8	
Control-2	11May2012	-632.09	0.78	974	0.29	988	191	18729	1.6	
16007-NCC	11May2012	-83.94	0.80	934	0.32	965	405	31491	2.6	
16007-1	11May2012	389.07	0.78	941	0.27	990	234	21559	1.7	
16007-2	11May2012	1487.80	0.73	971	0.30	988	209	18826	1.5	
PDK13-NCC	11May2012	-687.43	0.74	915	0.30	982	411	31585	2.5	
PDK13-1	11May2012	1521.96	0.74	956	0.31	986	204	19339	1.5	
PDK13-2	11May2012	-194.78	0.73	946	0.30	990	201	19151	1.5	

It is important to note that these values are restricted to the top 1000 expressed transcripts. **5'** and **3'** values are per-base coverage averaged across all top transcripts. 5' and 3' ends are 200 base pairs. Gap % is the total cumulative gap length divided by the total cumulative transcript lengths.

Mean Coverage

Low Expressed

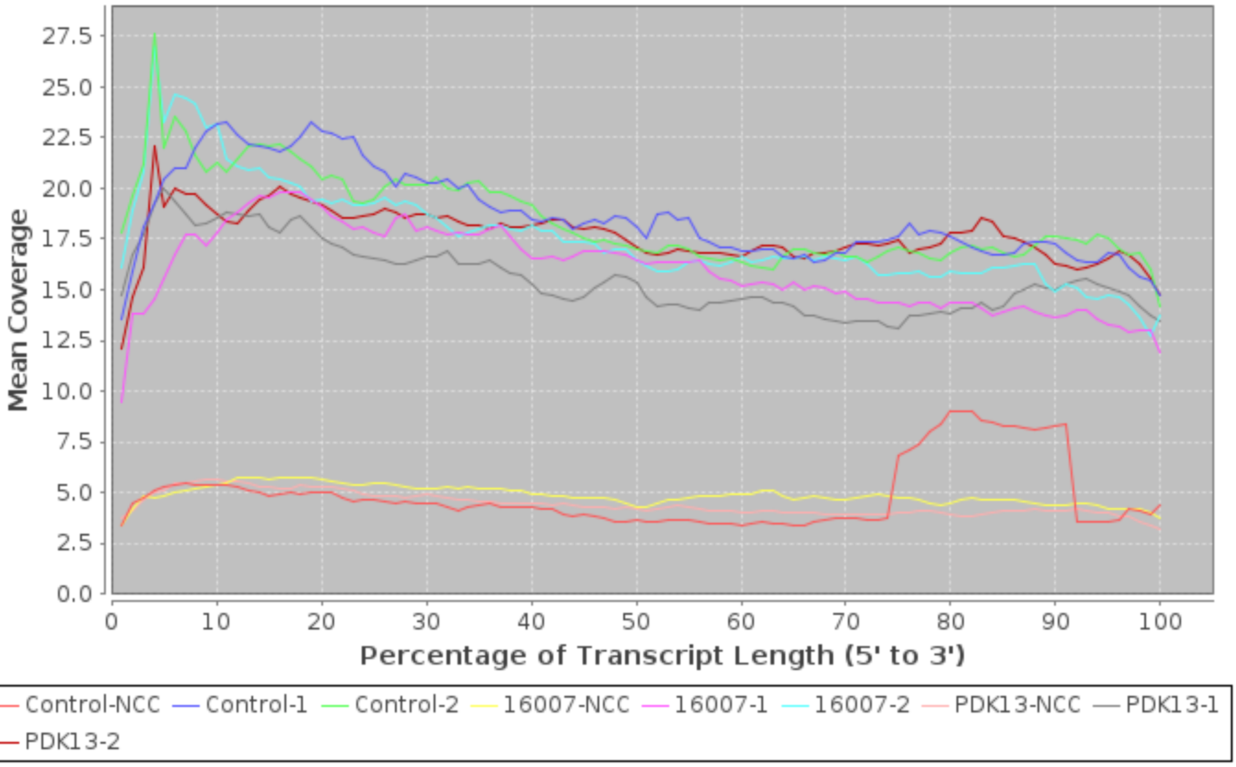

Medium Expressed

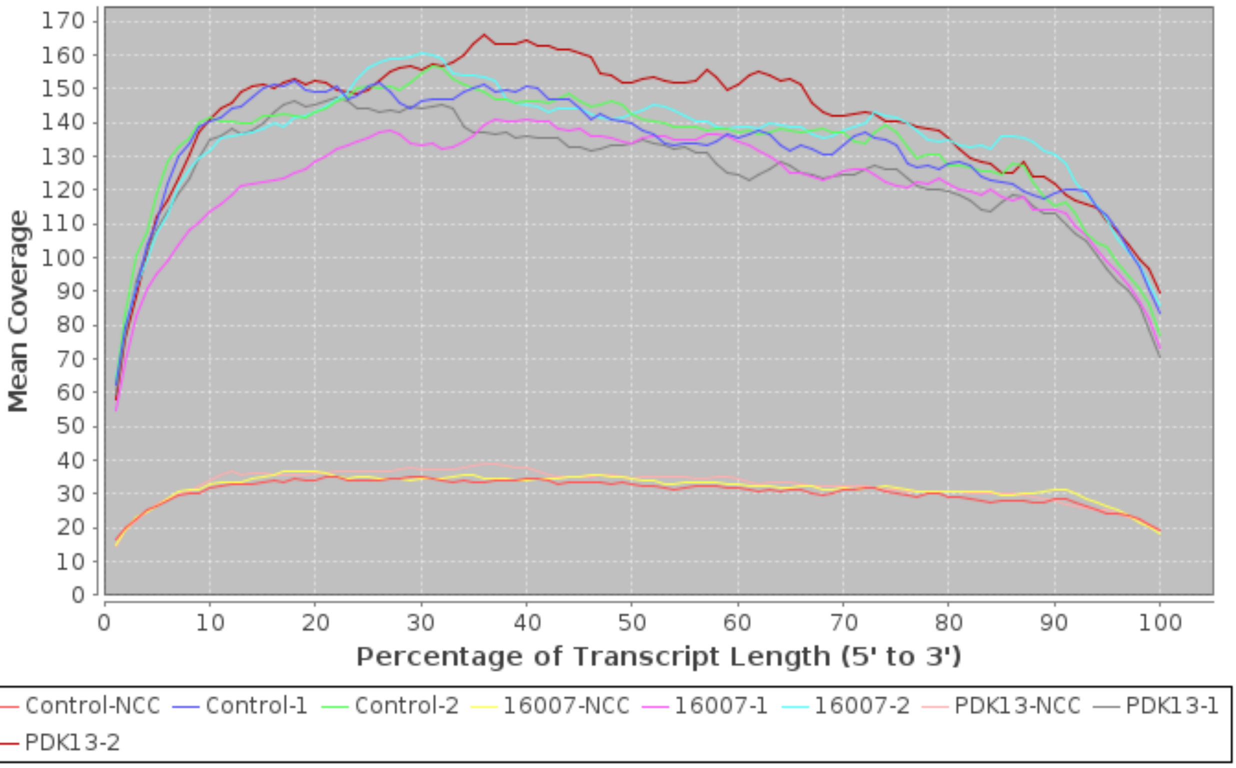

High Expressed

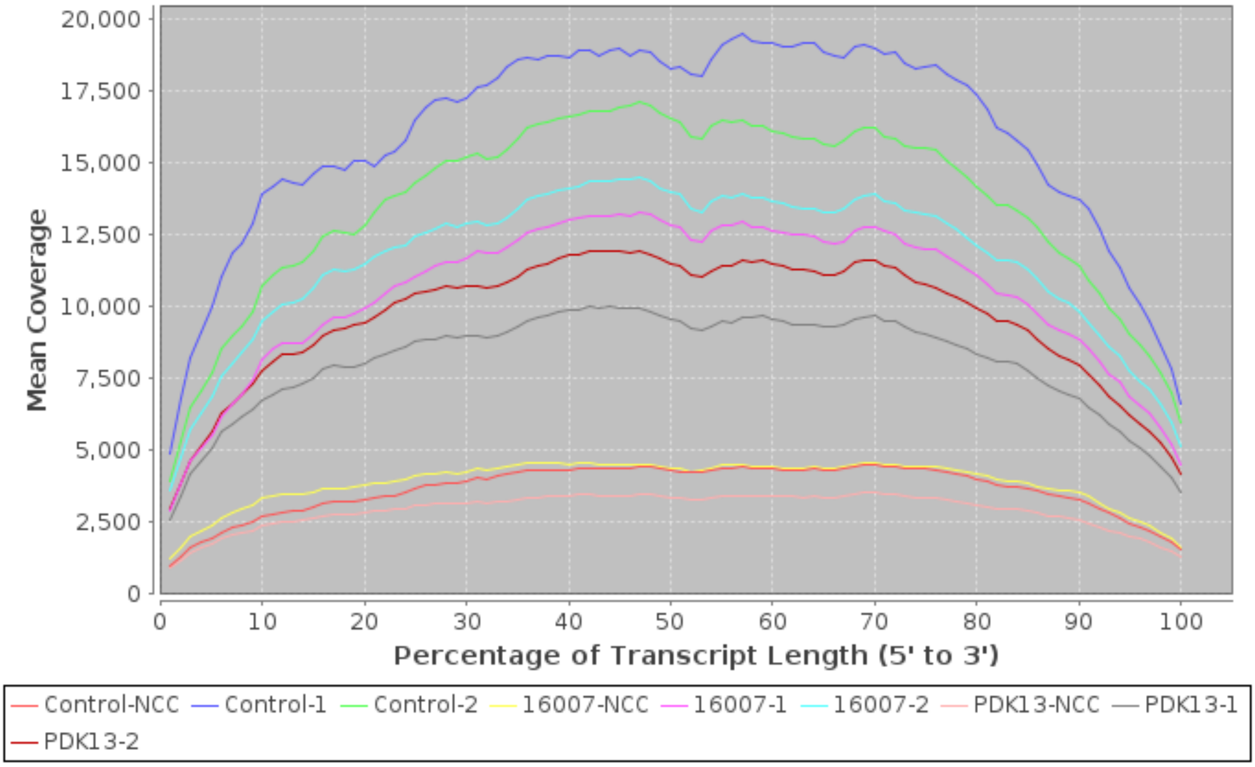

Mean Coverage from 3' End

Low Expressed

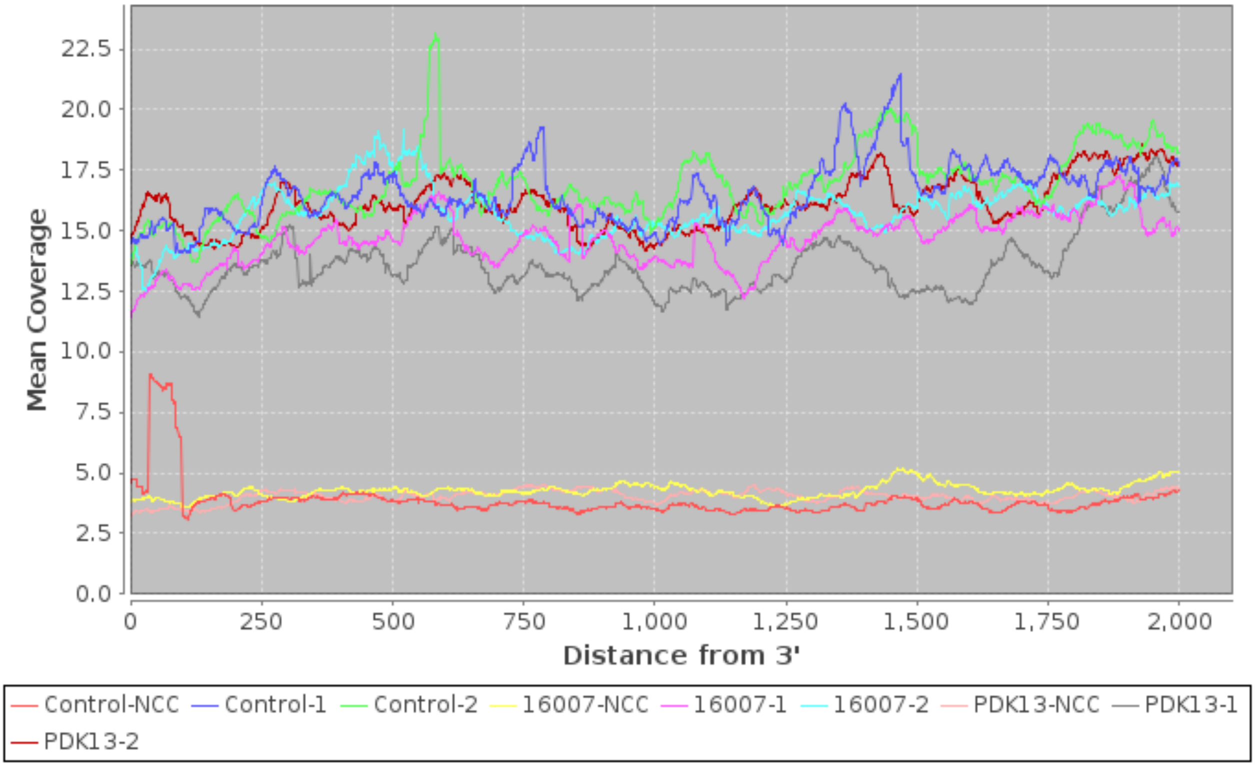

Medium Expressed

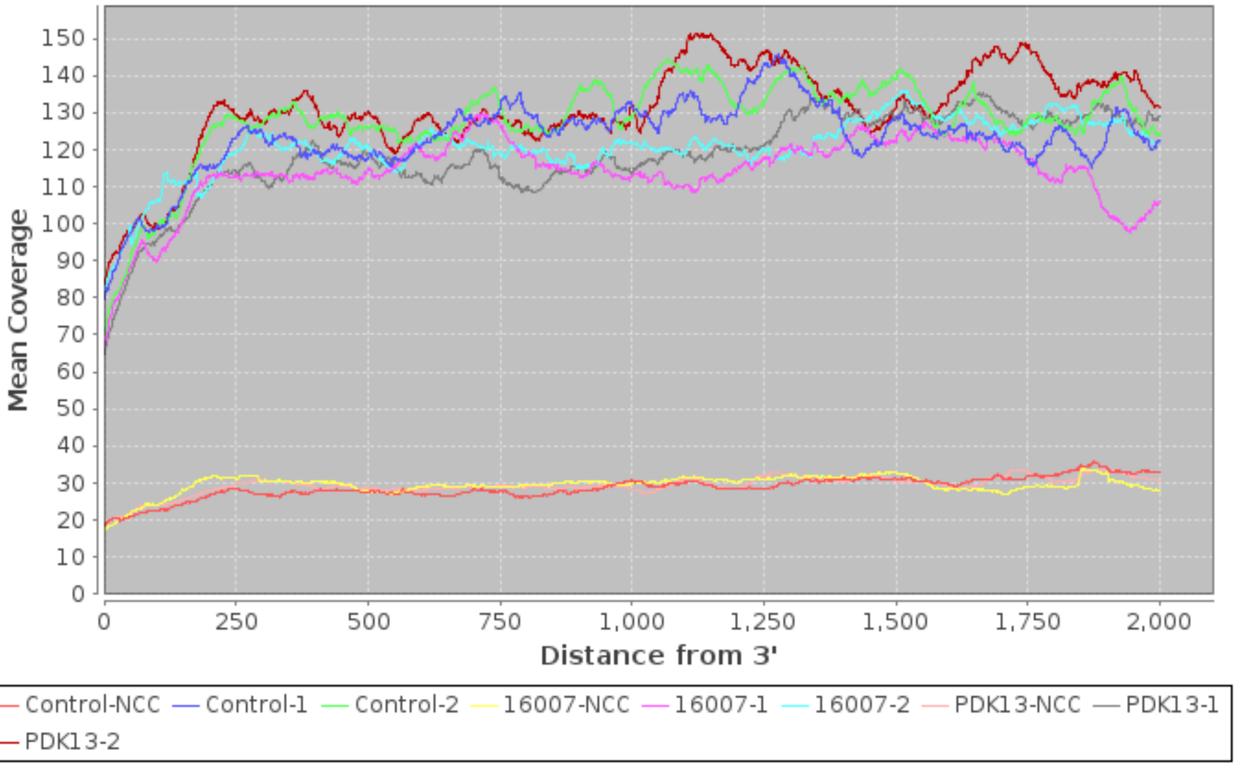

High Expressed

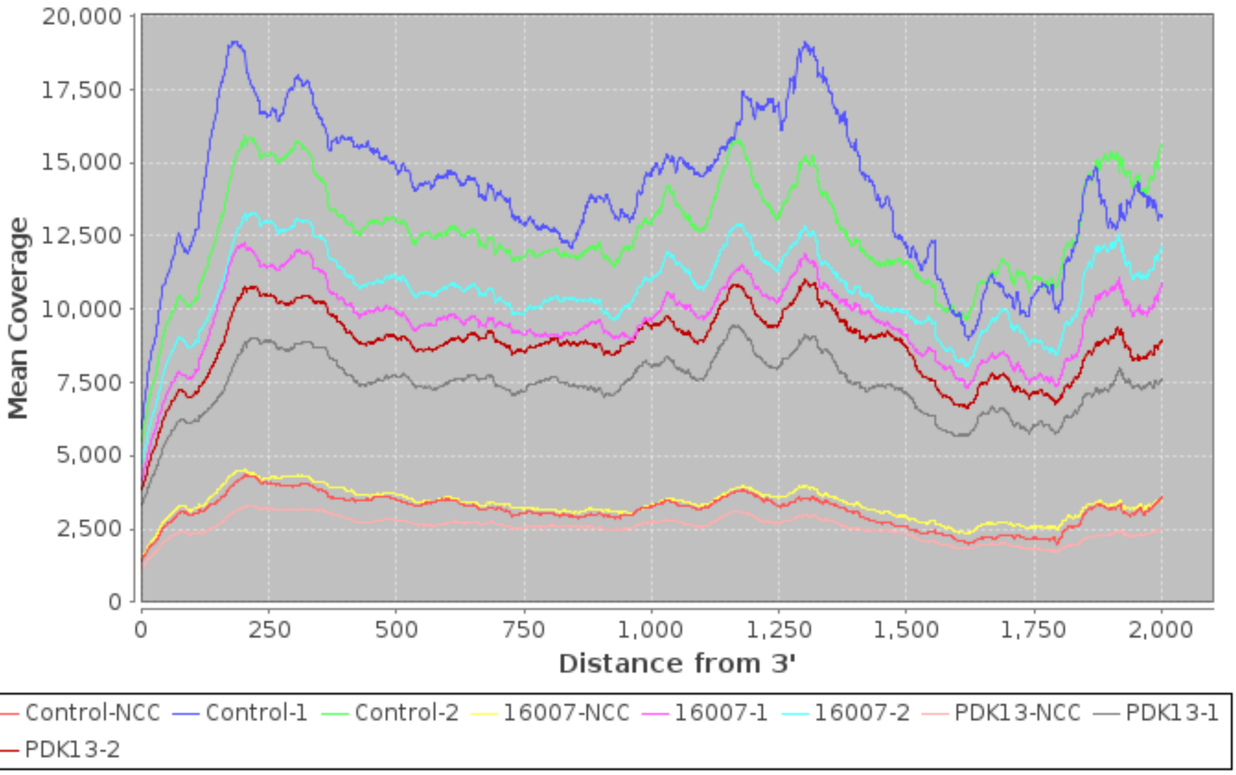

GC Stratification

- High GC
- Moderate GC
- Low GC

Files

File	Description
Metrics Tab Separated Value File	Text file containing all the metrics of the report in a single tab delimited file.
Mean Coverage Plot Data - Low Expr	Text file containing the data for mean coverage plot by position for low expression coverage
Mean Coverage Plot Data - Medium Expr	Text file containing the data for mean coverage plot by position for medium expression coverage
Mean Coverage Plot Data - High Expr	Text file containing the data for mean coverage plot by position for high expression coverage
Mean Coverage Plot Data - Low Expr	Text file containing the data for mean coverage plot by distance from 3' end for low expression coverage
Mean Coverage Plot Data - Medium Expr	Text file containing the data for mean coverage plot by distance from 3' end for medium expression coverage
Mean Coverage Plot Data - High Expr	Text file containing the data for mean coverage plot by distance from 3' end for high expression coverage
Mean Coverage Plot Data - Low Expr	Text file containing the data for gap length counts for low expression coverage
Mean Coverage Plot Data - Medium Expr	Text file containing the data for gap length counts for medium expression coverage
Mean Coverage Plot Data - High Expr	Text file containing the data for gap length counts for high expression coverage

Summary of Runtime Parameters

Option	Description	Value
Samples	Samples/Sample File used	BAM_Files-v5.list
Transcript Model	GTF formatted file containing the transcript definitions	hg19-ensembl_UCSC-corrected.gtf
Reference Genome	The genome version to which the BAM is aligned	hg19_Sorted.fa
Downsampling	For Coverage Metrics, the number of reads is randomly reduced to the given level	none
Detailed Report	The optional detailed report contains coverage metrics for every transcript	no details
rRNA Intervals	Genomic coordinates of rRNA loci	human_all_rRNA.fasta

Generated by RNA-SeQC v1.1.7 05/14/12

Run on Fri May 25 02:15:58 SGT 2012
